# Vaccine-Related Errors in Reconstitution in South Korea: A National Physicians’ and Nurses’ Survey

**DOI:** 10.3390/vaccines9020117

**Published:** 2021-02-02

**Authors:** Young Hwa Lee, Rebecca C. Harris, Hong Won Oh, Yongho Oh, Juan C. Vargas-Zambrano, Young June Choe

**Affiliations:** 1 College of Medicine, Hallym University, Chuncheon 24252, Korea; lkjin728@naver.com; 2 Sanofi Pasteur, 69007 Lyon, France; Rebecca.Harris@sanofi.com (R.C.H.); HongWon.Oh@sanofi.com (H.W.O.); YongHo.Oh@sanofi.com (Y.O.); Juan.Vargas@sanofi.com (J.C.V.-Z.); 3 Department of Infectious Disease Epidemiology, London School of Hygiene and Tropical Medicine, London WC1E 7HT, UK

**Keywords:** vaccine, vaccination error, reconstitution, prefilled syringe

## Abstract

Vaccine-related errors (VREs) result from mistakes in vaccine preparation, handling, storage, or administration. We aimed to assess physicians’ and nurses’ experiences of VREs in South Korea, focusing on reconstitution issues, and to understand the barriers to and facilitators of preventing them. This was a cross-sectional study using an internet-based survey to examine experiences of reconstitution-related errors, and experience or preference with regard to ready-to-use vaccines (RTU) by physicians and nurses. A total of 700 participants, including 250 physicians and 450 nurses, responded to the questionnaire. In total, 76.4% and 41.5% of the physicians and nurses, respectively, reported an error related to reconstituted vaccines. All errors had been reported as experienced by between 4.9% and 52.0% of physicians or nurses. The errors were reported to occur in more than one in 100 vaccinations for inadequate shaking of vaccines by 28.0% of physicians and 6.9% of nurses, incomplete aspiration of reconstitution vials by 28.0% of physicians and 6.4% of nurses, and spillage or leakage during reconstitution by 20.8% of physicians and 6.9% of nurses. A total of 94.8% of physicians had experience with RTU vaccines, and all preferred RTU formulations. In conclusion, this study highlights the high frequency and types of reconstitution-related errors in South Korea. RTU vaccines could help reduce the time needed for preparation and reduce the risk of errors in South Korea.

## 1. Introduction

Medication errors are an important preventable public health problem. The Institute of Medicine (IOM) report, To Err Is Human: Building a Safer Health System, identified medication errors as the most common type of error in healthcare [1]. Vaccine-related errors (VRE) result from mistakes in vaccine preparation, handling, storage, or administration. Such errors can result in adverse events or vaccine failure. They are preventable and detract from the overall benefits of the immunization program. The identification and correction of these incorrect immunization practices are of great importance.

To date, only a few previous studies have focused specifically on VREs. Findings have shown that approximately 10–35% of young children had at least one invalid dose administered [2,3]. When a multidisciplinary patient safety team was assembled to detect and analyze ambulatory medical errors by using a reporter-anonymous non-punitive process, a voluntary, team approach was effective in improving VRE reporting [4]. Several other studies have reported on specific VREs including extra immunization [5,6], improper route [7,8], and wrong drug administration [9]. Reconstitution-related VREs have been explored in several studies in Europe. A Belgian observational study and a European database study found 5-times and 2.5-times fewer mishandling or preparation errors when using fully-liquid ready-to-use hexavalent vaccines compared to reconstituted hexavalent vaccines [10,11]. In a physician’s survey in France, 28% reported occasionally omitting to reconstitute and 60% reported having not fully reconstituted vaccines [12]. However, almost all of the existing studies have been conducted in Europe; therefore, the Asian perspective on this issue is largely missing from the literature.

South Korea, with its 50 million population and annual birth-cohort of 300 thousand, has a strong immunization program that has both breadth and depth of coverage, and the government is looking to expand it further. South Korea is a high performer for immunization, reaching nearly 100% coverage for the majority of vaccines in its schedule [13]. Since the 2010s, the Korean immunization schedule has grown larger and more complex as new vaccines became available and recommendations expanded. Currently, several vaccines that require reconstitution are in use in South Korea. The national and local focus has now shifted to providing better vaccines with high-quality delivery to the population, with better safety and fewer VREs. No study exists to explore the frequency of reconstitution-related VREs in South Korea, or to explore what facilitators or barriers exist with regard to avoiding such VREs. This study aimed to assess physicians’ and nurses’ experiences of VREs in South Korea, focusing on reconstitution issues, and to understand the barriers to and facilitators of preventing such VREs.

## 2. Materials and Methods

### 2.1. Study Design and Participants

This was a cross-sectional study using an internet-based survey. For physicians, we conducted an online survey hosted by Medigate, a web-based physicians’ community with 115,976 registered physician members in South Korea (>95% of registered physicians), between 1 September 2020, and 14 September 2020. A convenience sample of physicians was recruited. Physicians could participate through a banner advertising the survey on the Medigate website and mobile application, through which registered members were directed to the questionnaire, until the sample/quota of 250 physicians meeting the inclusion criteria had been reached. Given nurses do not have Medigate access, a different recruitment method was required. To recruit participants among nurses, convenience sampling was conducted through posting on the bulletin boards of the webpages of the nurses groups at the Korean Nurses Association, Seoul National University Hospital, Catholic University of Korea Medical Center, and Children’s Hospital Association. In addition to convenience sampling, snowball sampling was also employed to ensure adequate recruitment; therefore those directly recruited could share the survey link with their peers by transmitting or retransmitting online messages. The first 450 respondents across the two recruitment methods were included. The nurses’ survey was conducted between 1 September 2020, and 6 November 2020. Inclusion criteria for this study were physicians or nurses aged at least 18 years who have ever prepared or used a reconstituted vaccine, and who prescribe or administer more than one vaccine per week on average for physicians, relaxed to more than one vaccine per month on average for nurses. Physicians were recruited whose major specialties were pediatrics, family medicine, and internal medicine, as these are the specializations who are most involved in vaccine administration in South Korea.

This was a descriptive study without comparative analysis between groups, therefore no formal sample size for comparative analysis was employed; however, a recruitment quota was selected for both doctors (*n* = 250) and nurses (*n* = 450), designed to be of a comparable size or larger than other similar studies in the literature (254–352 participants) [12,14,15]. The quota for nurses was larger to reflect the greater number of practicing nurses in Korea. Therefore, a convenience sample of a total of 700 healthcare providers, divided as 250 physicians and 450 nurses, was recruited. Recruitment was continued until the desired sample was achieved.

### 2.2. Survey

Informed consent was obtained from participants before enrolling in the survey. The survey included 24 questions, plus four initial questions to assess inclusion in the study. The questions covered age, gender, major practicing specialty, type of practice, geographic region, duration of professional experience, vaccination experience (weekly number of patients, weekly number of vaccinations, person participating in vaccine administration in practice, person participating in vaccine administration, etc.), experience of errors regarding reconstituted vaccines (type of error, frequency of an error occurring, cause of error, etc.), experience and preferences with regard to ready-to-use (RTU) vaccines (vaccines that are fully-liquid in a pre-filled syringe that require no reconstitution nor any other form of preparation; experience of using RTU vaccines, preference for RTU vaccines, reasons for preference, etc.).

### 2.3. Statistical Analysis

Frequencies and percentages were calculated for categorical and dichotomous variables. Means, standard deviations, and ranges were calculated for continuous variables. The population characteristics of participants were compared to the characteristics of the population from which they are sampled (physicians and nurses); the characteristics compared included age, gender, type of practice, geographic region, and years of practice. Comparison of responses of the first 50 respondents versus the last 50 respondents for primary outcomes were conducted to check for participation bias. Chi-square tests were used to examine sociodemographic characteristics, experience of error related problems regarding reconstituted vaccines, and experience or preference for RTU vaccines by profession, between physicians and nurses. A two-tailed *p*-value < 0.05 was considered statistically significant. SAS software version 9.4 (SAS Institute Inc., Cary, NC, USA) was used.

### 2.4. Ethics Review

This study was reviewed and approved by the Hallym University Institutional Board Review (IRB No: HIRB-2020-023).

## 3. Results

A total of 700 participants, including 250 physicians and 450 nurses, responded to the questionnaire. For the nurses, 30% (*n* = 133) were directly recruited, and 70% (*n* = 317) through snowballing. Table 1 shows the sociodemographic characteristics and current clinical practice profiles of participants. Of the respondents, 76% of physicians were men, whereas 95.3% of nurses were women. The proportion of male physician respondents is aligned with the physician population in Korea (74.6% male in 2017) [16], and the proportion of female nurses is aligned with the nursing gender balance (approx. 95% female) [17]. The mean age in years of physicians was 45.7 (95% CI: 44.7–46.8) and that of nurses was 34.8 (95% CI: 34.0–35.6), which were comparable to the national average age of physicians (44 years) and nurses (35 years old) [14]. In terms of practice capacity and vaccination practice, nurses tended to see and vaccinate more patients on a weekly basis than physicians, but the proportion of vaccines needing reconstitution was similar between the two groups. Finally, reported vaccination preparation and administration was different between the two groups, with vaccine preparation reported as conducted by physicians in 21.2% of physician responses, compared to 2% of nurse respondents. A similar trend was seen for administration, where 68% of doctors indicated they administer the vaccine themselves, while 16% of nurses reported physician-led administration.

The characteristics of the first 50 respondents were compared with the last 50 respondents to assess potential participation bias. No significant differences were identified in the physicians group. For nurses, there was no significant difference for most characteristics (gender, age, geographic region, major specialty, type of practice, vaccination-related practices such as number of patients and vaccines delivered), with the exception of the duration of professional experience, which was higher in the first 50 nurse respondents than the last 50 respondents: 88% of the first 50 reported at least 4 years of experience compared to 56% in the last 50 (*p* ≤ 0.01), likely resulting from the snowballing method. However, there was no significant difference for the proportion of nurses who worked less than 1 year (6% vs. 4%).

Table 2 summarizes physician and nurse reported errors or related problems regarding reconstituted vaccines. In total, 76.4% of the physicians reported experiencing an error related to reconstituted vaccines, compared to 41.5% of nurses (*p* < 0.0001). The different types of error were reported as having been experienced by between 15.6% and 52.0% of physicians and 4.9% to 19.1% of nurses. The most common errors reported by physicians were: inadequate shaking of the vaccine (51.6%), incomplete aspiration of the reconstitution vial (52.0%), and spillage or leakage during reconstitution (42.4%). Of all nurses, around a fifth reported inadequate shaking of the vaccine (19.1%). Other important reported errors that could cause the vaccine to be ineffective were forgetting to reconstitute (19.2% physicians and 7.6% nurses) or reconstituting with the wrong diluent (15.6% physicians and 4.9% nurses).

Nurses reported these errors less frequently, with only 17.3% and 48.7% reporting an error of at least 1 in 1000 vaccinations or none, compared to 20.8% and 8.8% for physicians (*p* < 0.0001). For nurses, 48.7% of the participants reported that an error had never occurred. The physicians responded that the main cause of errors was related to being less careful because of overall workload (57.5%), while the nurses indicated having a complex process in preparing the vaccine (44.6%) or having insufficient time to prepare the vaccine (42.4%).

In addition to describing the overall frequency of errors, participants were asked to estimate the frequency with which specific reconstitution-related errors occurred (Table 3). In general, errors were more frequently self-reported by physicians than nurses. The two most potentially impactful errors for vaccination, forgetting to reconstitute the vaccine and using the wrong diluent for reconstitution, were reported to occur in at least 1 per 100 vaccinations by 10.0% and 9.2% of physicians, respectively, while 2.7% and 1.6% of nurses reported this frequency of these errors, respectively.

When asked about the experience with existing RTU vaccines, most participants (94.8% of physicians and 85.6% of nurses) had used them before and most of them preferred RTU over vaccines that need reconstitution (100% physicians and 91.1% nurses) (see Table 4). There were differences in the reason for preference of RTU vaccines. Physicians preferences were mostly based upon reducing errors related to preparation (79.2%), followed by increasing work efficiency (71.6%). For nurses the most important reasons were related to increasing work efficiency (68.9%) and reducing preparation time (63.3%). Finally, almost one third mentioned that ultimately RTU would benefit patients.

## 4. Discussion

In this study, we found that a large proportion of physicians and nurses reported experiencing errors related to reconstituted vaccines, with three-quarters of physicians and almost half of nurses having experienced an error. All errors had been reported as being experienced between by 4.9% and 52.0% of physicians or nurses, occurring with a high frequency. The most frequently reported errors were inadequate shaking, incomplete aspiration of reconstitution vial, and spillage or leakage. Most physicians and nurses indicated preferences for RTU-formulated vaccines because these could decrease vaccine preparation time and vaccination-related errors.

Several vaccines are available in either RTU fully liquid formats or formats requiring reconstitution, which require different preparatory steps. Simplified vaccine preparation steps may save time and reduce potential VREs. The literature suggests that vaccines could be administered faster using a fully liquid vaccine compared to a vaccine requiring reconstitution [5]. One observational study in Belgium comparing hexavalent vaccines in these two formats found that preparation of a fully liquid vaccine can be completed in half the time necessary to prepare a non-fully liquid vaccine [10], and that five times fewer mishandling errors occurred when using a fully liquid RTU hexavalent vaccine instead of a non-fully liquid reconstituted hexavalent vaccine [10]. These errors included missed reconstitution, incomplete aspiration, loss of sterility due to touching of the rubber vial cap, and reuse of the same needle or needle twisting. Similarly, a European adverse events database reported 2.5-times fewer preparation errors with RTU than reconstituted hexavalent vaccines [11].

In our study, the most frequent type of error was inadequate shaking of the vaccine, closely followed by incomplete aspiration of the reconstitution vial and spillage or leakage during reconstitution. A survey of more than 300 physicians in France found that 28% reported occasional omission of reconstitution of pentavalent or hexavalent vaccines, and 60% did not fully reconstitute the vaccine [12]. Almost all of the existing studies have been conducted in Europe; therefore, the Asian perspective on this issue had largely been missing from the literature. Our study in South Korea found a slightly lower proportion reporting having forgotten reconstitution (19.6% of physicians and 10.7% of nurses), but many other reconstitution-related errors were also reported, totaling 76.4% of physicians and 41.5% of nurses reporting having experienced some kind of reconstitution error. This body of evidence suggests that fully liquid vaccines may save time and optimize VRE reduction essential for patient safety and vaccine effectiveness.

The results of our study are in line with reports elsewhere, demonstrating the high frequency of reported reconstitution-related errors, and goes further to demonstrate the frequency by type of error and by profession. Providing a safe vaccination practice poses a significant logistical issue but is important in providing quality care to patients. To protect patients from vaccine-related errors, clinicians need information to direct improvement efforts. Our data demonstrate that eight different reconstitution-related errors occur, with inadequate shaking, incomplete aspiration and spillage or leakage as the most experienced errors. Only approximately half of participants reported always preparing a new vaccine when reconstitution errors occurred, and more than a quarter reported that they never prepared a new vaccine following an error. Therefore, encouraging preparation of new vaccine where appropriate could help reduce any potential impacts on vaccine efficacy. However, this does create vaccine wastage, so corrective actions to avoid the error would be better from a health system perspective. Such errors could be managed through improved awareness and training to reduce the frequency of these errors, or where available through using RTU formulations to avoid the reconstitution errors completely. Various risk-reduction strategies, such as training healthcare professionals, affixing storage bin labels, redesigning labeling, and packaging of vaccines may be proposed; however, an RTU formulation of the vaccine may allow for safer vaccination practice in most settings.

We also found that physicians reported more frequent errors than nurses, including reconstitution with incorrect diluent and loss of equipment for reconstitution. Nurses reported needle twisting or spillage more frequently than physicians. The difference may be attributable to the setting of the practice in South Korea, where primary care access is easy in terms of proximity and cost, which results in a high patient burden per clinic potentially leading to increased risk of errors [18]. There may be a correlation between patient volume and VREs among physicians in primary care. In the primary care setting, physicians may not be able to reduce the probability of VREs if they feel fatigued during working hours, whereas shifting schedule of the nurses may reduce this risk. Nurses, on the other hand, take in charge of initial process in the vaccine preparation in most instances, therefore experience more programmatic errors than the physicians. As such, our findings suggest that broad coverage of corrective activities to reduce errors could be of use, potentially including some physician-focused activities to reduce frequency of errors experienced by physicians. Alternatively, replacement of reconstitution-requiring vaccines with RTU-based vaccines may provide advantages by preventing reconstitution-related errors in various clinical settings [19]. Moreover, most physicians and nurses preferred a vaccine with an RTU formulation. For physicians, this was reported as a preference due to reduction in VREs and streamlining of the overall processes, including reducing vaccine preparation time and increasing work efficiency. For nurses, RTU vaccines may decrease the overall work process, thus increasing work efficiency. This is in line with findings from a recent survey from Italy, showing that healthcare personnel prefer a vaccine that can reduce the time needed for preparation, while reducing the risk of errors as much as possible [15].

This study is limited by the survey’s cross-sectional nature, and we were unable to determine the potential direction of the cause–effect relationship for the associations observed. The convenience sampling method may have introduced some selection and participation bias. Furthermore, for nurses the dual-method convenience sampling and snowballing might increase the risk of such bias. To minimize the impact of selection bias, internet communities with the broadest accessibility to the physician and nurse populations were selected. Comparison of the recruited population to the overall nurse and physician populations in South Korea demonstrated similar characteristics, suggesting minimal bias. However, a predominance of physicians aged in their 40s and nurses in their 30s, might limit generalizability to the most junior or senior physicians and nurses. A within-sample comparison was also made between the first 50 and last 50 respondents to assess potential participation bias. This found no difference in the physician group, and for nurses the only difference was in the length of professional experience, yet other parameters were comparable between the groups. As the last 50 were less experienced, it suggests that the snowball method may have been biased towards more newly qualified nurses, thus this group might be overrepresented in the sample compared to the general nurses in South Korea. Nonetheless we do not believe as this would change the results significantly as they reported delivery of a high volume of vaccinations and use of reconstituted vaccines. Differences in the reported proportion of preparation and administration by each type of healthcare professionals was indicative of potential participation or selection bias, favoring those actively involved in vaccination activities. However, given the research question and inclusion criteria of the study, this was anticipated and was considered indicative of appropriate study design for the research question. Overall, although participation bias cannot be excluded, these analyses suggest that such bias is likely minimal, therefore the results are considered valid for physicians and nurses actively involved in vaccination activities. This study is one of the largest studies of its kind, and to our knowledge is the first study to explore the types and frequency of reconstitution-related vaccine errors in Asia, and therefore has important implications for vaccination practice in South Korea and similar settings in East Asia.

## 5. Conclusions

In conclusion, this study showed a high frequency and various types of reconstitution-related errors in South Korea. RTU vaccines could help reduce the time needed for preparation and reduce the risk of errors in South Korea.

## Figures and Tables

**Table 1:** Sociodemographic and current clinical practice profile of participants by profession.

Characteristics	Physician (*n* = 250)	Nurse (*n* = 450)
Gender, *n* (%)		
Men	190 (76.0)	21 (4.7)
Women	60 (24.0)	429 (95.3)
Age, mean (95% CI)	45.7 (44.7–46.8)	34.8 (34.0–35.6)
Median (Interquartile Range)	45 (40–50)	33 (28–40)
Geographic region		
Metropolitan city	144 (57.6)	295 (65.6)
Provincial city	95 (38.0)	153 (34.0)
Town	11 (4.4)	2 (0.4)
Duration of professional experience, *n* (%)		
<1 years	1 (0.4)	16 (3.6)
1–3 years	16 (6.4)	104 (23.1)
≥4 years	233 (93.2)	330 (73.3)
Type of practice, *n* (%)		
Individual	68 (27.2)	111(24.7)
Group	30 (12.0)	17(3.8)
Independent within delivery hospital	1 (0.4)	42(9.3)
Paid (clinic/children’s hospital/general hospital/delivery hospital)	151 (60.4)	267(59.3)
Healthcare center	0 (0.00)	13(2.9)
Practice capacity and vaccination practice		
Number of patients at the practice per week, mean (95% CI)	296 (274–318)	400 (267–534)
Weekly number of vaccinations, mean (95% CI)	46 (40–53)	115 (39–191)
Proportion of vaccinations requiring reconstitution, mean (95% CI)	34 (31–36)	29 (26–32)
Proportion of vaccinations for children (<18 years old), mean (95% CI)	58 (53–62)	27 (24–30)
Person participates in vaccine preparation in practice, *n* (%)		
Physician	53 (21.2)	9 (2.0)
RN	109 (43.6)	343 (76.2)
AN	88 (35.2)	98 (21.8)
Person participates in vaccine administration in practice, *n* (%)		
Physician	170 (68.0)	74 (16.4)
RN	80 (32.0)	376 (83.6)
AN	0 (0.00)	0 (0.00)
Respondent’s participation in vaccine delivery activities, *n* (%)		
Vaccine preparation	62 (24.8)	200 (44.4)
Vaccine administration	153 (61.2)	129 (28.7)
Vaccine preparation and administration	116 (46.4)	324 (72.0)
Other	17 (6.8)	15 (3.3)

Abbreviations: CI, confidence interval; AN, assistant nurse; RN registered nurse.

**Table 2:** Reported error or related problems regarding reconstituted vaccines by profession.

Errors	Physician (*n* = 250)	Nurse (*n* = 450)	*p*-Value
Experienced an error related to reconstituted vaccines, *n* (%)			
Yes	191 (76.4)	187 (41.5)	<0.0001
No	37 (14.8)	170 (37.8)	
Uncertain	22 (8.8)	93 (20.7)	
Type of error, *n* (%) (multiple selections)			
Inadequate shaking of vaccine	129 (51.6)	86 (19.1)	-
Incomplete aspiration of the reconstitution vial	130 (52.0)	66 (14.7)	
Spillage or leakage during reconstitution	106 (42.4)	66 (14.7)	
Needle twisted when inserted in vial stopper	74 (29.6)	53 (11.8)	
Same needle used for reconstitution and injection	58 (23.2)	45 (10.0)	
Forgetting to reconstitute the vaccine	48 (19.2)	34 (7.6)	
Loss of equipment for reconstitution	40 (16.0)	28 (6.2)	
Reconstitution with incorrect diluent	39 (15.6)	22 (4.9)	
Frequency of an error occurring, *n* (%)			
Never	22 (8.8)	219 (48.7)	<0.0001
<one in 1000 vaccinations	52 (20.8)	78 (17.3)	
One in 1000 vaccinations	27 (10.8)	41 (9.1)	
One in 500 vaccinations	48 (19.2)	35 (7.8)	
One in 100 vaccinations	72 (28.8)	43 (9.6)	
>one in 100 vaccinations	29 (11.6)	34 (7.6)	
Cause of error, *n* (%) (multiple selections)			
Being less careful because of overall workload	132 (57.5)	59 (25.5)	-
Having insufficient time to prepare the vaccine	77 (33.8)	98 (42.4)	
Paying less attention to complete dissolution	77 (33.8)	63 (27.3)	
Having complex process in preparing of the vaccine	62 (27.2)	103 (44.6)	
Frequency of preparation of new vaccine when an error occurs, *n* * (%)	228 (100.0)	231 (100.0)	
Never	67 (29.4)	57 (24.7)	0.1193
25% of the time	37 (16.2)	28 (12.1)	
50% of the time	14 (6.1)	10 (4.3)	
75% of the time	6 (2.6)	14 (6.1)	
Always	104 (45.6)	122 (52.8)	

*n* *: number of respondents who experienced an error.

**Table 3:** Types of error and reported frequency of each error occurring.

Type of Error, N (% for Each Rows)		Frequency of Errors (% of All Participants)			
		≤One in 1000 Vaccinations	One in 500 Vaccinations	≥One in 100 Vaccinations	Total Experiencing Error
Physician (*n*=250)	Inadequate shaking of vaccine	29(11.6)	30(12.0)	70(28.0)	129(51.6)
	Incomplete aspiration of the reconstitution vial	31(12.4)	29(11.6)	70(28.0)	130(52.0)
	Spillage or leakage during reconstitution	28(11.2)	26(10.4)	52(20.8)	106(42.4)
	Needle twisted when inserted in vial stopper	18(7.2)	17(6.8)	39(15.6)	74(29.6)
	Same needle used for reconstitution and injection	18(7.2)	10(4.0)	30(12.0)	58(23.2)
	Forgetting to reconstitute the vaccine	16(6.4)	7(2.8)	25(10.0)	48(19.2)
	Loss of equipment for reconstitution	10(4.0)	3(1.2)	27(10.8)	40(16.0)
	Reconstitution with incorrect diluent	11(4.4)	5(2.0)	23(9.2)	39(15.6)
Nurse (*n*=450)	Inadequate shaking of vaccine	42(9.3)	13(2.9)	31(6.9)	86(19.1)
	Incomplete aspiration of the reconstitution vial	25(5.6)	12(2.7)	29(6.4)	66(14.7)
	Spillage or leakage during reconstitution	22(4.9)	13(2.9)	31(6.9)	66(14.7)
	Needle twisted when inserted in vial stopper	15(3.3)	14(3.1)	24(5.3)	53(11.8)
	Same needle used for reconstitution and injection	29(6.4)	4(0.9)	12(2.7)	45(10.0)
	Forgetting to reconstitute the vaccine	16(3.6)	6(1.3)	12(2.7)	34(7.6)
	Loss of equipment for reconstitution	9(2.0)	5(1.1)	14(3.1)	28(6.2)
	Reconstitution with incorrect diluent	13(2.9)	2(0.4)	7(1.6)	22(4.9)

**Table 4:** Experience or preference for a vaccine with a ready-to-use (RTU) feature by profession.

Experience or Preference for RUT Vaccines	Physician	Nurse
	(*n* = 250)	(*n* = 450)
Experienced in using RTU vaccines, *n* (%)	237 (94.8)	385 (85.6)
Preference for a vaccine with RTU formulation, *n* (%)*	250 (100.0)	410 (91.1)
Could decrease vaccination-related error compared to reconstitution vaccines	198 (79.2)	109 (24.2)
Could decrease vaccine preparation time	198 (79.2)	285 (63.3)
Could increase work efficiency	179 (71.6)	310 (68.9)
Would decrease overall work process	114 (45.6)	287 (36.8)
Would benefit patients	81 (32.4)	149 (33.1)
Other	3 (1.2)	5 (1.1)

*n* *: number of respondents who preferred vaccines with an RTU formulation.

## Data Availability

The data presented in this study are available on request from the corresponding author.
